# The Effectiveness of a Dietary Supplement with Honey, Propolis, *Pelargonium sidoides* Extract, and Zinc in Children Affected by Acute Tonsillopharyngitis: An Open, Randomized, and Controlled Trial

**DOI:** 10.3390/ph17060804

**Published:** 2024-06-19

**Authors:** Fabio Cardinale, Dionisio Franco Barattini, Valentina Martinucci, Maria Morariu Bordea, Luca Barattini, Serban Rosu

**Affiliations:** 1 Complex Operating Unit Paediatrics, Giovanni XXIII Paediatric Hospital, University of Bari, 70124 Bari, Italy; 2 Opera Contract Research Organization, Tigermed Company, 300209 Timisoara, Romania; barattini@operacro.com; 3 Pediatrica S.r.l., 57121 Livorno, Italy; rd@pediatrica.it; 4 Cabinet Medical Medicina de Familie Dr Morariu Bordea, 300425 Timisoara, Romania; cabinetmbmorariu@yahoo.com; 5 Tigermed Italy S.r.l., 16129 Genova, Italy; personal@lucabarattini.com; 6 Department of Oral and Cranio-Maxillo-Facial Surgery, University of Medicine and Pharmacy Victor Babes, 300041 Timisoara, Romania; serbanrosu@gmail.com

**Keywords:** dietary supplements, tonsillitis, pharyngitis, nasopharyngitis, honey, propolis, zinc, *Pelargonium sidoides*, severity score

## Abstract

Physicians are currently finding products for pediatric respiratory diseases of viral etiology to reduce the inappropriate use of antibiotic therapy. This study evaluated PediaFlù (Pediatrica S.r.l.), a dietary supplement already on the market composed of honey, propolis, *Pelargonium sidoides* extract, and zinc (DSHPP), in children affected by acute tonsillopharyngitis (ATR). The open-label, randomized, and controlled study compared DSHPP + standard of care (SoC) versus SoC alone for six days. Children between 3 and 10 years with an ATR ≤ 48 h, a negative rapid test for beta-hemolytic *Streptococcus*, or a culture identification of nasal and/or pharyngeal exudates were included. A tonsillitis severity score (TSS) and the number of treatment failures (using ibuprofen or high-dose paracetamol as rescue medication) were the primary endpoints. DSHPP+ SoC showed better performance than SoC alone for TSS sub-scores: throat pain and erythema on day 6 (*p* < 0.001 and *p* < 0.05), swallowing (*p* < 0.01 on day 4), and TSS total score on days 4 and 6 (*p* < 0.05 and *p* < 0.001). Only one patient (SoC group) had treatment failure for ibuprofen administration. No adverse events were reported. DSHPP is an optimal adjuvant in the treatment of URTI and could potentially be useful in the daily clinical practice of paediatricians evaluating the correct antibiotic prescription.

## 1. Introduction

Acute tonsillopharyngitis (ATR), which is typically caused by viruses, is a common reason for consultation with pediatricians and general practitioners (GPs). In contrast, bacterial etiology requires antibiotic therapy and is present in approximately 30% of all cases of ATR. Therefore, in the great majority of cases, ATR does not require antibiotic treatment [1]. Furthermore, guidelines [2] and a recent review [3] emphasize that antibiotics should not be applied in the pediatric population for apparent viral respiratory illnesses (sinusitis, pharyngitis, and bronchitis). Instead, they should be administered only to children with pharyngitis clearly caused by group A *Streptococcus*. Even when considering other bacterial infections that require antibiotic treatment, the discrepancy between the median incidence of *Streptococcal pharyngitis*, which is 1764 per 100,000 children [4], and the annual rate of antibiotic prescribing for childhood upper respiratory tract infections (URTI), which is 421 prescriptions per 100,000 population in the US [5], is evident. This explains the observation made by Fleming-Dutra KE that one-third of outpatient antibiotic prescriptions for pediatric URTI are inappropriate [6]. Consequently, research and clinical practice are currently exploring the potential benefits of products that can be used in pediatric respiratory diseases of viral etiology, with the aim of reducing the inappropriate use of antibiotic therapy.

In light of the aforementioned considerations, Pediatrica S.r.l. (Livorno, Italy) has developed a dietary supplement (DS) in an oral solution, PediaFlù^®^ (DSHPP), designed for pediatric use as an adjuvant for respiratory tract health and well-being. The composition of this product is based on the synergistic action of honey and propolis, which have been demonstrated to have beneficial effects on the respiratory tract [7,8]. The DSHPP also contains *Pelargonium sidoides* extract (Pelagon P 70™) and zinc. Six clinical trials using honey in a total of nearly 900 children were analyzed in the Cochrane meta-analysis published on 2018 [9]. The analysis revealed that honey was associated with favorable outcomes in terms of cough frequency, patient quality of life, and the quality of sleep for both parents and children. The limitations of the evaluated studies included the small sample sizes, the heterogeneous administration of the tested product (different types of honey were used in different studies), and the short duration of treatment (often one night only). Nevertheless, the meta-analysis concluded that honey alleviated cough symptoms compared with no treatment or diphenhydramine but was not found to be more effective than dextromethorphan. The beneficial activity of honey has been linked to the reduction of free radicals produced at the site of inflammation and its capacity to reduce the use of antibiotics. Additionally, it has been demonstrated to enhance the immune system, promoting the proliferation of human peripheral blood B cells, neutrophils, and T lymphocytes [10,11]. A recent pragmatic randomized clinical trial (RCT) conducted in Spain evaluated the efficacy of honey in reducing cough symptoms in adult patients with acute bronchitis. Although the trial did not reach its original enrollment goal of 668 patients [12], it did demonstrate that the duration of severe-to-moderate cough in patients treated with 30 mg of honey for two weeks was comparable to that of patients in the other arms (dextromethorphan 15 mg t.i.d. and ipratropium bromide inhaler 20 μg 2 puffs t.i.d.). A systematic review demonstrated that propolis exerts a beneficial effect on glutathione, glutathione peroxidase (GPX), and total antioxidant capacity (TAC) levels and has been shown to be safe for use as a dietary supplement. Moreover, it can be employed as an adjuvant treatment in diseases where oxidative stress plays a crucial role in the pathogenesis [13]. A number of studies have demonstrated that the administration of propolis to patients diagnosed with respiratory disorders has resulted in improvements in symptoms. In an RCT with a double-blind design, propolis was found to produce a statistically significant reduction in the number of days required for symptom resolution in both bacterial and viral URTI [14]. The findings of this study are consistent with those of a prior case-control investigation conducted on children regarding the treatment of rhinopharyngitis [15]. Propolis has also demonstrated advantages in asthmatic patients, including increased forced vital capacity (FVC), pulmonary forced expiratory volume in the first second (FEV1), and peak expiratory flow rate (PEFR) [16]. A statistically significant reduction in the incidence of exacerbations and an improvement in symptoms and quality of life of patients affected by chronic obstructive pulmonary disease (COPD) was evidenced in RCTs using a combination of propolis and N-acetylcysteine (NAC) [17,18]. A systematic review [19] examined data from 8526 patients who received zinc supplementation, demonstrating a reduction in the duration of the common cold. As evidenced by the scientific literature, the extract of *Pelargonium sidoides* may be an effective treatment for disorders affecting the respiratory tract, particularly in pediatric populations with acute non-streptococcal tonsillopharyngitis [20,21]. These beneficial effects of *Pelargonium sidoides* are the result of reducing harmful inflammation [22] from one side and enhancing the immune response by increasing phagocytic activity, oxidative burst, intracellular killing of pathogens, release of nitric oxide (NO) in macrophages, and release of tumor necrosis factor (TNF)-α, and interleukin (IL)-1 and IL-12 [23]. 

The data provided above indicate the effectiveness of the honey, propolis, zinc, and *Pelargonium sidoides* extract components when administered separately. Published reviews and the daily clinical practice of thousands of pediatricians have also emphasized the safety of these components when used in combination with other products. Therefore, integrating the four components into a single DSHPP has the advantage of activating the synergic action of honey and propolis [7,8]. It also provides medical doctors with the opportunity of administering a single, effective, and safe product in conjunction with the traditional standard of care (SoC).

The present clinical trial was planned to confirm the results observed in clinical practice. The objective was to assess whether a six-day administration of the investigational DSHPP, in conjunction with SoC, could result in a more favorable ATR symptomatology compared to patients receiving SoC alone. The confirmation of this hypothesis would support the candidacy of DSHPP as an effective tool for pediatricians and general practitioners.

## 2. Results

### 2.1. Patient Disposition

**Figure 1:**
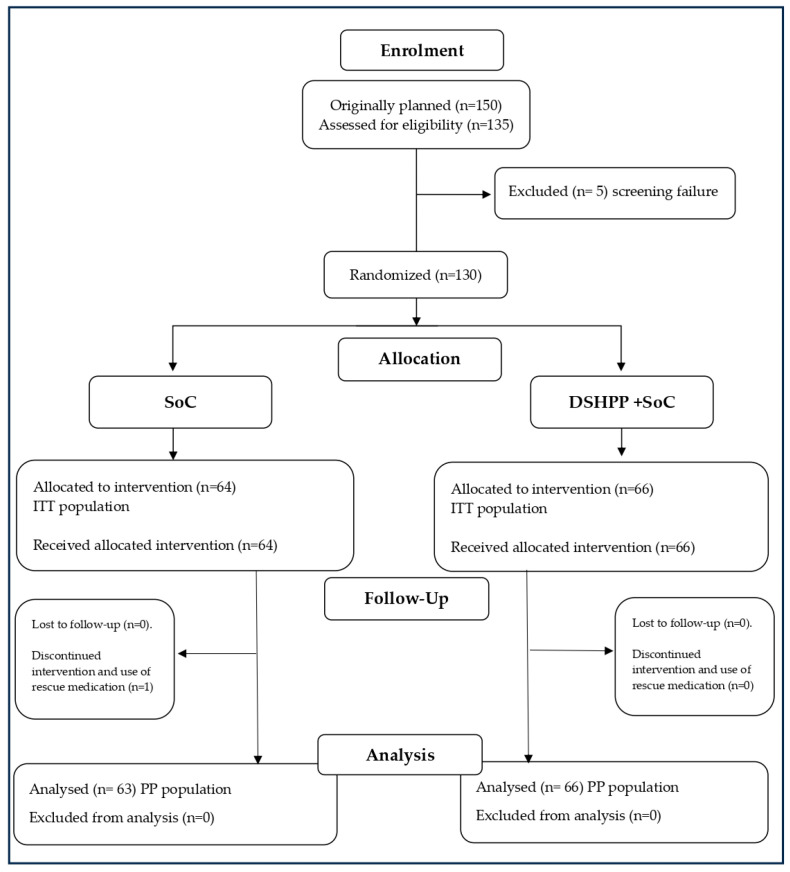
Flow diagram of the study. Standard of care (SoC); DSHPP dietary supplement PediaFlù^®^ based on honey and propolis associated to zinc and *Pelargonium sidoides* extract.

The enrollment period began on 3 June 2021, and concluded on 6 August 2021. The study was terminated on 12 August 2021 (last patient and last visit). A total of 135 individuals were initially screened, and 130 of them were deemed eligible for participation in the trial. The children were randomly assigned to either the DSHPP + SoC group (n = 66) or the SoC alone group (n = 64). The intention-to-treat (ITT) population included all 130 subjects who were enrolled and completed the study. The per protocol (PP) population consisted of 129 patients, as one child from the DSHPP + SoC group was excluded from the analysis due to discontinuation of treatment and withdrawal from the study following the use of rescue medication (Figure 1). 

The patient characteristics outlined in Table 1 showed no significant differences in baseline demographic and anthropometric variables between the DSHPP + SoC and SoC alone groups (*p* > 0.05, Mann–Whitney U test). 

### 2.2. Administered Treatments and Compliance 

All patients were fully compliant with the administration of DSHPP and SoC (low-dosage paracetamol and benzydamine hydrochloride). There were no significant differences in the number of doses and quantity of benzydamine hydrochloride administered between the groups. By contrast, there is a statistically significant difference (*p* < 0.01, Mann–Whitney U test) in the mean (SD) of administered doses of low-dosage paracetamol: 13.67 (4.34) and 15.92 (3.02) in DSHPP + SoC and SoC, respectively.

### 2.3. Efficacy

#### 2.3.1. Primary Outcomes

##### Tonsillitis Severity Score

The primary efficacy outcome was the change in the tonsillitis severity score (TSS) from baseline to the final visit, analyzed separately for total score and for the five sub-scores: throat pain, difficulty in swallowing, salivation, erythema, and fever. The randomization between groups was executed properly (M diff. = +0.3, U = 1762, *p* = 0.091, CI95: 8.88–9.32). The balance between groups was maintained at the outset of medication and dietary supplement administration (M diff. = +0.3, U = 1763, *p* = 0.114, and CI95: 8.88–9.32).

##### TSS Total Score

The total score for TSS during the study period indicated that children treated with DSHPP + SoC exhibited a lower rating than children in the SoC alone group (Table 2). In particular, the difference between the two groups was statistically significant at days 4 and 6, with *p*-values of 0.034 and 0.002, respectively (Mann–Whitney U test).

##### TSS Throat Pain Sub-Score

**Figure 2:**
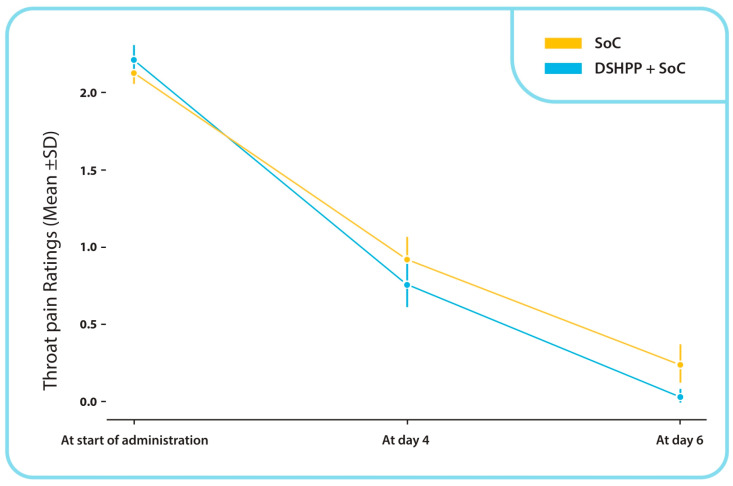
Change in TSS Throat pain; mean ± SD ratings from start of administration to final visit. On day 6 *p* < 0.001 (Mann–Whitney U test).

Two of the 66 subjects (3.03%) from the DSHPP + SoC group reported experiencing throat pain at the final visit. In contrast, 14 of the 63 subjects (23.33%) from the SoC alone group had throat pain on day 6. Consequently, a statistically significant lower number of subjects from the DSHPP + SoC group compared to the SoC alone group were experiencing throat pain at the final visit (chi-square test, *p* < 0.001). Figure 2 illustrates the change in TSS throat pain ratings from baseline to final visit. It shows a mean (SD) reduction in the DSHPP + SoC group from 2.2 (0.4) to 0.0 (0.2), while the SoC alone group exhibited a mean (SD) slight increase from 2.1 (0.3) to 0.2 (0.5). The statistical analysis revealed a significant difference between the two groups (*p* < 0.001, Mann–Whitney U test).

##### TSS Difficulty in Swallowing Sub-Score

**Figure 3:**
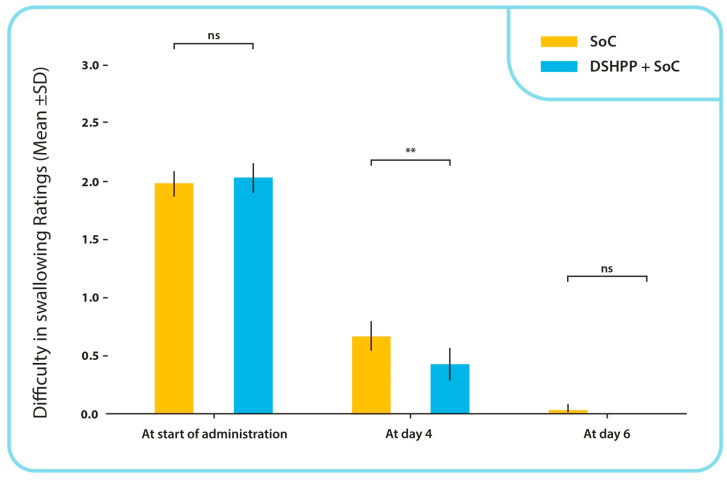
Difficulty in swallowing mean ± SD ratings with independent statistical tests between administration groups, from start of administration to final visit. ** *p* < 0.01; ns: non-significance.

At the final visit, none of the subjects in the DSHPP + SoC group exhibited any difficulty swallowing. In contrast, two of the sixty-three subjects in the SoC alone group (3.17%) reported difficulties swallowing at the final visit (day 6). The difference between the two groups was not statistically significant at the final visit, as determined by a chi-square test (*p* = 0.146). Figure 3 presents the comparison of the means (SD) between administration groups for difficulty in swallowing ratings from the beginning of administration to the final visit. A statistically significant difference was observed only on visit day 4 (*p* < 0.01, Mann–Whitney U test).

##### TSS Salivation and Fever Sub-Score

The statistical analysis of these two parameters during the course of the study showed no statistically significant difference between the group treated with DSHPP + SoC and the group treated with SoC alone.

##### TSS Erythema Sub-Score

**Figure 4:**
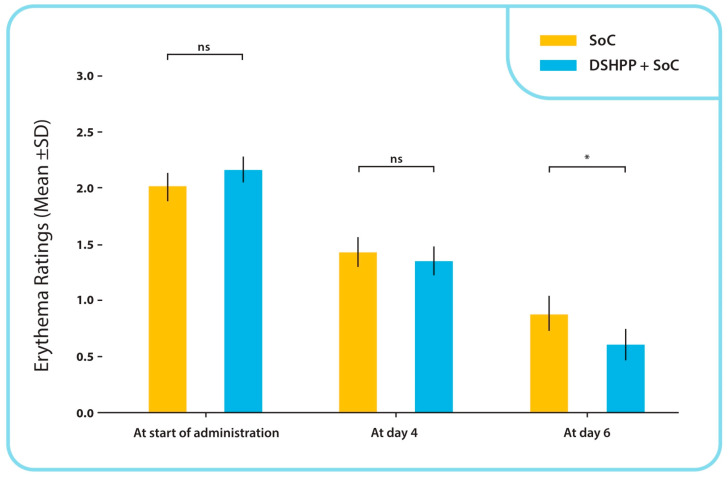
Erythema mean ± SD ratings from start of administration to final visit with chi-square test. * *p* < 0.05; ns: non-significance.

The balance between groups was not altered at baseline or at the start of the administration, indicating that the randomization between groups was properly executed (*p* = 0.065, Mann–Whitney U test). At the final visit, 46 of 63 subjects (73.0%) from the SoC group and 36 of the 66 subjects (54.5%) in the DSHPP + SoC group reported erythema. Figure 4 demonstrated that the difference between the two groups was statistically significant (*p* = 0.030, chi-square test).

##### Use of Rescue Medications

The additional primary outcome was the use of rescue medications (ibuprofen and paracetamol above the limit as defined in the protocol of 30 mg/kg/dose) in the two groups and the resulting treatment failure. Only one patient in the SoC alone group received ibuprofen (1.6% of patients in the SoC alone group). The paracetamol doses administered to patients in both groups during the study were all below the threshold of 30 mg/kg/dose. The mean (SD) paracetamol dose in the DSHPP + SoC group was 12.52 (2.83) mg/kg/dose, while the mean (SD) paracetamol dose in the SoC group was 12.79 (2.53) mg/kg/dose.

#### 2.3.2. Secondary Outcomes

The secondary efficacy outcomes were the investigator global assessment (IGA) and the patient global assessment (PGA). A comparison was conducted for each of the parameters between the two groups at the final visit (Day 6).

##### IGA

Table 3 presents the IGA between groups on day 6. All subjects from the DSHPP + SoC group experienced high levels of treatment efficacy, with 83.3% rating it as very good and 16.7% rating it as good. Subjects from the SoC alone group rated the treatment as very good (58.7%), good (28.6%), and moderate (12.7%). The difference between the two groups was statistically significant (*p* = 0.04, chi-square test).

##### PGA

The results of the PGA ratings indicate that none of the subjects in the DSHPP + SoC group reported poor treatment efficacy. In contrast, three (4.8%) subjects from the SoC alone group reported poor treatment efficacy. A statistically significant number of subjects (*p* = 0.013, chi square test) in the DSHPP + SoC group demonstrated superior treatment efficacy in comparison to those in the SoC alone group (Table 4).

### 2.4. Safety

In both the DSHPP + SoC group and the SoC alone group, there were no adverse events (AEs) or serious adverse events (SAEs) reported by any children during the trial. Furthermore, a global evaluation of the safety of the treatment was conducted by the investigator at the conclusion of the study (Day 6). The ratings for the DSHPP + SoC group were 93.9% very good and 6.1% good, while the ratings for the SoC alone group were 55.6% very good and 30.2% good. The investigators did not record any concomitant medication.

## 3. Discussion

Our team has been trying for years to have a judicious antibiotic prescription for children with URTI [24]. In fact, we defined the child population in which we would avoid antibiotic prescriptions in our daily clinical practice as pediatricians, following international guidelines [24,25] that indicated stringent diagnostic criteria, weighing the benefits and harms of antibiotic therapy. The criteria we followed included as follows:

(1) ATR should be diagnosed early (possibly in the last 48 h) through clinical evaluation of symptoms, including sore throat and catarrhal angina.
(2) A negative result from a rapid test for group A beta-hemolytic *Streptococcus* (GABHS) or culture and the identification of nasal and/or pharyngeal exudate tests were mandatory.
(3) The severity of ATR should be clearly defined with the exclusion of cases with lacunar or follicular angina, indications for antibiotic therapy (i.e., abscess and septic tonsillitis), immunodeficiencies, and chronic illnesses.
(4) The exclusion of children under 3 years of age. This point was added because of the potential difficulty in justifying the exclusion of antibiotic therapy with the parents of infants of this age.

In general, the international guidelines [24,25] were characterized as follows:

- The target was the medical doctor visiting and treating children affected by URTI.
- The objective was to modify therapeutic strategies based on antibiotic prescription that have been proved to be not justified in the majority of cases
- The final result should be that thousands of medical doctors understand situations when antibiotics may not be indicated, and this should reduce the consumption of antibiotics inadequately administered to children.

Despite the efforts of scientific associations and institutions, incorrect antibiotic administration for URTI in children remains higher than the recommended benchmarks because of inappropriate prescribing habits [26]. In recent years, the medical literature has proposed a number of alternative strategies for achieving judicious antibiotic prescription in children with URTI. Some of these [27,28] involved Internet-based communication skills training in healthcare settings, which again were aimed solely at the prescribing physician. They obtained discrete success in reducing inappropriate antibiotic prescribing in adults, although they were not able to change the prescribing reality of antibiotic therapy in childhood URTIs. Other authors have based their work on the assumption that clinical uncertainty in primary care regarding the prognosis of children with respiratory tract infections contributes to the unnecessary use of antibiotics. Their aim was to reduce the misuse of antibiotic therapy in URTIs by improving the correct information aimed at the prescribing physician and focusing their task on equipping the physician with functional prognostic tools. An example of this approach was the RCT named CHICO, conducted with 294 GPs in the UK. The trial aimed to improve the early identification of children at low risk of future hospitalization to reduce clinical uncertainty [29]. The program utilized a web-based system with a prognostic algorithm to promptly identify children with acute cough and respiratory tract infections at low, average, or elevated risk of hospitalization in the next 30 days, accompanied by prescription guidance. The intervention was well received by GPs, but it did not alter prescribing habits, and antibiotic prescription rates remained largely unchanged. Kronman et al. [30] used a different approach, implementing the Dialogue Around Respiratory Illness Treatment (DART) quality improvement program. It was hypothesized that pairing Internet-based communication skills training with individualized antibiotic prescription audits and feedback would result in improved outcomes from 2015 to 2018 in 19 pediatric practices. The observed outcome (a 7% reduction in the probability of antibiotic prescription) is relatively modest, particularly when one considers the extensive resources deployed and the data regarding antibiotic prescription for *Streptococcal pharyngitis* (aRR 0.66; 95% CI, 0.50–0.87). 

Finally, we believe that while communication, training, and information can be useful tools for improving the therapeutic strategies of physicians and increasing their prognostic ability, they are insufficient on their own. These approaches were designed to address the prescribing physicians alone. In our view, they should also engage the parents of children and even consider (in association with the traditional standard of care) the administration of new adjuvant tools that are accepted by both physicians and parents. Therefore, when the therapeutic strategy chosen by the clinician is not to use antibiotic therapy after performing a rapid test for GABHS, we believe that it is appropriate to flank the SoC already known to parents with other products that have been shown in clinical trials to have adjuvant action in total safety. The coordination carried out by the University of Timisoara (Romania) for the entire duration of the study and the supervision of Prof. Fabio Cardinale (Italy) were the fundamental key elements characterizing our research. The tested product (DSHPP) also has the advantage of being made up of ingredients such as honey and propolis, which are often used by parents themselves outside of prescription for their beneficial properties on the respiratory system. This can be an added advantage and ensure the maintenance of compliance, which is a prerequisite for the quick resolution of a child’s disease.

## 4. Materials and Methods

### 4.1. Study Design

This multicenter, controlled, open-label, and randomized parallel-group superiority trial was conducted at three Romanian centers: Cabinet Medical Dr. Maria Morariu Bordea (Timișoara, Romania), Cabinet Medical Dr. Dorina Herteg (Timișoara), and Cabinet Medical Dr. Cristian Radu Matei (Otelu Rosu). The project team consisted of a scientific supervisor of the study, investigators, and was coordinated by the Academic Research Organization Department of Clinical Trials of the University of Medicine and Pharmacy Victor Babes. The contract research organization (CRO), Opera CRO, a Tigermed company based in Timișoara, Romania, was selected by the project team to manage the logistics and administration of the study. This included tasks such as protocol submission, project management, site monitoring, data management, and statistical analysis.

The project team created the study protocol following the methodology described by Fabio Cardinale et al., 2023 [31], and in accordance with the CONSORT-C Statement [32], which was specifically tailored for a trial involving a pediatric population. The study was registered at ClinicalTrials.gov (NCT04899401) and the overview of study methods (including ethics, design, randomization and allocation, setting, patient recruitment, inclusion and exclusion criteria, intervention and comparator, sample size calculation and statistical methods) was detailed in a recent publication [33].

Prior to the commencement of the trial, the protocol and related documents were submitted to the Romanian National Agency for Medicines and Medical Devices (ANSM) and were also approved by the local ethics committee (EC) of the involved centers (on 23 April and 27 April 2021).

The randomization procedure was executed through an internet-based platform provided by the CRO, accessible at all times. Patients were randomly assigned to either the control group, which received standard of care (SoC) alone, or the interventional group, which received DSHPP in addition to SoC. A non-involved staff member was responsible for carrying out the allocation process, without any input from the patients themselves. It should be noted that the patients’ parents were not blinded to the randomization process.

### 4.2. Study Population

Only participants who were accompanied by their parents or caregivers to clinical sites from 6 June 2021 (start of enrollment) to 31 August 2021 (end of enrollment of 130 children) were eligible for the study. No additional recruitment efforts were made through the use of flyers, social media advertisements, or other promotional methods. The enrolled children must have been affected by ATR with a TSS score of at least 8 for no more than 48 h. They must also have had negative results on rapid tests for group A beta-haemolytic *Streptococcus* (GABHS) or negative culture and identification of nasal and/or pharyngeal exudates, and negative Severe Acute Respiratory Syndrome Coronavirus 2 (SARS-CoV-2) infection. Table 5 outlines the remaining inclusion and exclusion criteria.

### 4.3. Intervention Population

#### 4.3.1. Tested Product: DSHPP

The DSHPP tested is a dietary supplement already on the market in Italy as PediaFlù (Pediatrica Srl, Livorno, Italy). The formulation includes honey (5.5 g/100 mL), PropolNext Plus (equivalent to propolis, 7.7 mg/100 mL), Pelagon P-70 (equivalent to *Pelargonium sidoides*, 133.3 mg/100 mL), and zinc (13.3 mg/100 mL). DSHPP is supplied in the form of an oral solution. In the study DSHPP was administer it orally, at a dose of 5 mL, three times a day, for children under the age of 6 for a period of 6 days, while children over the age of 6 should take 10 mL, three times a day, for a period of 6 days. Due to concerns for patient safety, the investigator was authorized to discontinue the administration of DSHPP at any time and prescribe alternative treatments as needed for the patient’s well-being.

#### 4.3.2. Comparator: SoC

All patients included in the study were administered the SoC. This de facto included children assigned to the control group (who received SoC alone) and children assigned to the interventional group (who received SoC in addition to DSHPP).

The following products and treatments were administered as SoC:

- Nasopharyngeal lavage through the administration of hydrating fluids to facilitate the elimination of body fluids, along with the aspiration of secretions, saline solution for nasal irrigation and nasal sprays containing seawater.
- Paracetamol (acetaminophen) (120 mg/5 mL): as antipyretic (fever is defined as body temperature > 38.5 °C), as needed, 10 mg/kg/dose. The dosage was to be administered every 6 to 8 h, according to the leaflet. The maximum dosage was 30 mg/kg/day.
- Benzydamine hydrochloride was to be administered by throat sprays for 6 days in children under 6 years of age; the dosage was to be 1 spray per 4 kg of body weight, up to a maximum of 4 sprays at one time, 2 to 6 times daily. In children from 6 to 12 years, the benzydamine hydrochloride dosage was to be 4 sprays, administered two to six times daily. Each spray corresponds to 0.17 mL of solution.

#### 4.3.3. Rescue Medication and Treatment Not Permitted

The administration of coumarin-based products and antibiotics was not permitted during the study.

Ibuprofen and paracetamol were the only permitted treatments in cases of necessity as rescue medications for the relief of severe symptoms of ATR. The following is a summary of the permitted treatments:

ibuprofen (100 mg/5 mL) orally;
high dose (>30 mg/kg/day) paracetamol (120 mg/5 mL) administered orally.

The use of rescue medication to a child was automatically classified as treatment failure.

### 4.4. Statistical Methods

#### 4.4.1. Trial Hypothesis

We formulated a superiority hypothesis to assess whether symptoms in patients with ATR can be improved by a six-day course of DSHPP plus SoC compared with SoC alone. The primary hypothesis was that the minimal clinical difference between the test group (DSHPP + SoC) and the control group (SoC alone) should result in a two-point decrease in mean TSS after six days of treatment. The null hypothesis was that treatment with DSHPP + SoC would not result in a significant improvement in the symptoms of the treated patients compared to the control group (treated with SoC alone). 

#### 4.4.2. Sample Size Calculation 

The sample size was determined based on the results of a prior study [21] that measured TSS as the primary outcome. The required sample size was calculated using the formula for comparing two means (two-sample) at a 5% significance level, 80% power, and a minimum clinically significant difference of 2 (SD 3.85 points). This indicated that 120 patients (130 with the estimated dropout) were required. To achieve this target, it was estimated that approximately 150 children should be screened.

#### 4.4.3. Statistical Analyses

Categorical variables were described using frequencies and percentages, and comparative analyses were performed using the chi-squared test. Quantitative variables, if normally distributed, were described by means and standard deviations; non-normally distributed variables were described by medians and interquartile ranges. The Student’s *t*-test and Mann–Whitney U test were used to perform comparative analyses according to the distribution of these variables. Factorial analysis of variance was employed to evaluate any interactions between quantitative variables and linear progression models, with the objective of identifying and addressing any potential confounding biases associated with the independent variables.

### 4.5. Evaluation Outcomes 

#### 4.5.1. Primary Outcomes

The primary efficacy outcomes were the change in TSS and the number of treatment failures between the two groups.

The TSS comprised five sub-scores: throat pain, difficulty in swallowing, salivation, pharyngeal erythema, and fever. The investigator evaluated and scored TSS at each visit as follows: throat pain, difficulty in swallowing, salivation, and pharyngeal erythema were rated on a scale of severe (3), moderate (2), mild (1), or absent (0), respectively. The investigator evaluated fever based on the following scale: The temperature scale is as follows: 37.5 °C to <38.5 °C = 1, 38.5 °C to <39.5 °C = 2, and ≥39.5 °C = 3. The results were compared in terms of the absolute change in the total score and for all sub-scores from baseline to the final visit.

The number of treatment failures was calculated by comparing the administration of rescue medications (ibuprofen and paracetamol > 30 mg/kg/dose) between the two groups.

#### 4.5.2. Secondary Outcomes

The secondary efficacy outcomes were the investigator global assessment (IGA) and the patient global assessment (PGA). For both, the comparison was between groups at the end of the study period (Day 6). The investigator used a four-point scale, ranging from 1 (excellent) to 4 (poor), to assess the IGA. In contrast, the patient global assessment (PGA) was rated by patients on a 5-point scale, where 1 was defined as “very satisfied” and 5 as “very unsatisfied”.

#### 4.5.3. Safety

Any AE and SAE were recorded during the study to evaluate the incidence of AE/SAE. Furthermore, the investigators conducted a comprehensive assessment of the safety of the treatments using a four-point scale (1 = very good safety, 2 = good safety, 3 = moderate safety, and 4 = poor safety), with a comparison between groups at the conclusion of the trial.

#### 4.5.4. Schedule of Examinations

Prior to initiating the study, the investigators informed each patient and their parent or legal guardian of the purpose and nature of the study, the potential risks and benefits, and requested consent by requiring them to sign an ICF. Furthermore, investigators provided the children with instructions on how to correctly respond to the tests, with a particular focus on the PGA. Finally, parents were requested to maintain a temperature log and report the usage of rescue medication in the patient diary during the study. Furthermore, it was emphasized that any unused medication be returned at the conclusion of the study and that the investigator be contacted in the event of an adverse event.

Table 6 outlines the schedule of examinations and procedures to be performed during the study period.

## 5. Conclusions

The main limitation of this study is its open-label and controlled rather than double-blind design. This choice is motivated by logistical and economic reasons. 

It is essential to highlight that our objective in this study was not merely to demonstrate that DSHPP associated with SoC will result in a clinical benefit. It was also crucial to prove that this administration was accompanied by optimal DSHPP compliance and safety, which are both essential requirements for a product to be administered to children. For this reason, our research hypothesis of associating the use of DSHPP with SoC may represent a potential alternative in the first-line treatment of acute respiratory tract infections, beyond the strict indication for antibiotic treatment, and can contribute to improving the judicious antibiotic prescription for children with URTI.

## Figures and Tables

**Table 1:** Per protocol (PP) population: demographics of the patients at the baseline visit by assignment group. ns: non-significant.

Characteristics		All no. = 129	DSHPP + SoC no. = 66	SoC no. = 63	*p*-Value
Age (years)	Mean (SD)	5.52 (2.07)	5.8 (2.01)	5.22 (2.11)	ns
	Median	5	5.5	5	
	Range	3–10	3–10	3–10	
BMI (kg/m^2^)	Mean (SD)	16.41 (3.06)	16.71 (3.0)	16.1 (3.11)	ns
	Median	15.54	15.97	15.42	
	Range	10.42–30.26	10.42–25.69	11.76–30.26	
Height (cm)	Mean (SD)	114.51 (14.95)	116.56 (15.36)	112.37 (14.31)	ns
	Median	114	116	108	
	Range	89–160	89–160	90–148	
Weight (kg)	Mean (SD)	22.1 (8.29)	23.32 (8.74)	20.83 (7.66)	ns
	Median	20	22	19	
	Range	11–60	12–60	11–54	

**Table 2:** Tonsillitis severity score (TSS) total score mean ±SD ratings between administration groups, on day 0, day 4, and day 6 with Mann–Whitney U test. ** *p* < 0.01; * *p* < 0.05; ns: non-significant.

		DSHPP + SoC	SoC	*p*-Value
Start of administration	no.	66	63	
	Mean (SD)	9.2 (1.4)	8.9 (1.2)	ns
Day 4	no.	66	63	
	Mean (SD)	3.2 (1.4)	3.8 (1.7)	*
Day 6	no.	66	63	
	Mean (SD)	0.7 (0.7)	1.2 (1.0)	**

**Table 3:** Investigator global assessment (IGA) ratings in the two groups at final visit. *p*-values correspond to chi square tests.

IGA	Grade	DSHPP + SoC	SoC	*p*-Value
Subjects no. (%)		66 (100.0%)	63 (100.0%)	
	Very Good	55 (83.3%)	37 (58.7%)	0.040
	Good	11 (16.7%)	18 (28.6%)	
	Moderate	0 (0.0%)	8 (12.7%)	
	Poor	0 (0.0%)	0 (0.0%)	

**Table 4:** Patient global assessment (PGA) in the two groups at final visit. *p*-values correspond to chi square tests.

PGA	Grade	DSHPP + SoC	SoC	*p*-Value
Subjects no. (%)		66 (100.0%)	63 (100.0%)	
	Very Good	53 (80.3%)	35 (55.6%)	0.013
	Good	12 (18.2%)	19 (30.2%)	
	Moderate	1 (1.5%)	6 (9.5%)	
	Poor	0 (0.0%)	3 (4.8%)	

**Table 5:** Study inclusion and exclusion criteria.

Inclusion Criteria	
1	Male and female (children aged 3 to 10 years).
2	Acute tonsillopharyngitis/rhinopharyngitis (ATR; sore throat, catarrhal angina), duration of symptoms ≤ 48 h.
3	Negative with rapid test for group A beta-hemolytic *Streptococcus* (GABHS) or culture and identification of nasal and/or pharyngeal exudates; negative for SARS-CoV-2 infection.
4	Tonsillitis symptom score (TSS) ≥ 8 points.
5	Both parents are willing to provide written informed consent prior to participation in the clinical trial.
6	Children older than 6 years must also have the ability and willingness to provide written informed consent.
Exclusion criteria	
1	Evidence of lacunar or follicular angina.
2	More than 2 past episodes of tonsillitis in the previous 12 months.
3	Mandatory indication for antibiotic therapy (e.g., abscess, septic tonsillitis, status postrheumatic fever, poststreptococcal glomerulonephritis, and minor Sydenham chorea).
4	Treatment with antibiotics within 4 months prior to study enrollment.
5	Hemorrhagic diathesis increases and chronic illnesses (e.g., severe heart, kidney, or liver disease, and primary or secondary immunodeficiencies).
6	Close contact history with individuals infected with SARS-CoV-2 within 10 days before showing symptoms.
7	Known or suspected allergic reactions to any study medication.
8	Concomitant therapy that may affect study results or have known interactions with study medications (such as coumarin derivatives).
9	Participation in another clinical trial within 3 months before enrollment.

Children who met all the study requirements were randomly assigned to either the DSHPP + SoC or SoC alone treatment arm and provided with the necessary products to complete the study. Parents were also instructed on how to follow the assigned treatment plan. Patients (by their parents or tutors) were permitted to withdraw from the trial at any time without providing a reason.

**Table 6:** Study schedule of examinations and procedures.

Time Point	Visit 1	Visit 2	Visit 3	Visit 4
	Day −2 to −1	Day 0	Day 4	Day 6
Informed consent	✓			
Inclusion criteria	✓			
Exclusion criteria	✓	✓	✓	✓
Demographics and medical history	✓			
Physical examination	✓	✓	✓	✓
Disease assessment	✓	✓	✓	✓
Rapid test for GABHS ^a^ or nasal and/or pharyngeal exudate culture plus SARS-CoV-2	✓			
Concomitant treatments	✓	✓	✓	✓
TSS ^b^	✓	✓	✓	✓
Product delivery		✓		
Product return				✓
Subject diary delivery		✓		
Patient’s diary verification			✓	
Subject diary return				✓
Products accountability				✓
IGA ^c^				✓
PGA ^d^				✓
Global assessment of the safety				✓
Adverse events	✓	✓	✓	✓

^a^ GABHS: group A beta-hemolytic *Streptococcus*. ^b^ TSS: tonsillitis symptom score. ^c^ IGA: investigator global assessment. ^d^ PGA: patient global assessment.

## Data Availability

The full protocol of the study has been deposited at Protocols.io [29]. The data sets analyzed for this study are available from the corresponding author upon request. The detailed description of the study protocol was recently published [31].

## References

[1] Pelucchi C., Grigoryan L., Galeone C., Esposito S., Huovinen P., Little P., Verheij T., ESCMID Sore Throat Guideline Group (2012). Guideline for the management of acute sore throat. Clin. Microbiol. Infect..

[2] Hersh A.L., Jackson M.A., Hicks L.A., American Academy of Pediatrics Committee on Infectious Diseases (2013). Principles of judicious antibiotic prescribing for upper respiratory tract infections in pediatrics. Pediatrics.

[3] Vicentini C., Vola L., Previti C., Brescia V., Dal Mas F., Zotti C.M., Bert F. (2022). Antimicrobial Stewardship Strategies Including Point-of-Care Testing (POCT) for Pediatric Patients with Upper-Respiratory-Tract Infections in Primary Care: A Systematic Review of Economic Evaluations. Antibiotics.

[4] Yildiz I., Gonullu E., Soysal A., Oner C.N., Karabocuoglu M. (2023). The Epidemiology of Influenza Virus Infection and Group A Streptococcal Pharyngitis in Children between 2011 and 2018 in an Outpatient Pediatric Clinic. Cureus.

[5] Hersh A.L., Shapiro D.J., Pavia A.T., Shah S.S. (2011). Antibiotic prescribing in ambulatory pediatrics in the United States. Pediatrics.

[6] Fleming-Dutra K.E., Hersh A.L., Shapiro D.J., Bartoces M., Enns E.A., File T.M., Finkelstein J.A., Gerber J.S., Hyun D.Y., Linder J.A. (2016). Prevalence of inappropriate antibiotic prescriptions among US ambulatory care visits, 2010–2011. JAMA.

[7] Al-Waili N., Al-Ghamdi A., Ansari M.J., Al-Attal Y., Salom K. (2012). Synergistic effects of honey and propolis toward drug multi-resistant *Staphylococcus aureus*, *Escherichia coli* and *Candida albicans* isolates in single and polymicrobial cultures. Int. J. Med. Sci..

[8] Dimitriu L., Constantinescu-Aruxandei D., Preda D., Nichițean A.-L., Nicolae C.-A., Faraon V.A., Ghiurea M., Ganciarov M., Băbeanu N.E., Oancea F. (2022). Honey and Its Biomimetic Deep Eutectic Solvent Modulate the Antioxidant Activity of Polyphenols. Antioxidants.

[9] Oduwole O., Udoh E.E., Oyo-Ita A., Meremikwu M.M. (2018). Honey for acute cough in children. Cochrane Database Syst. Rev..

[10] Abuharfeil N., Al-Oran R., Abo-Shehada M. (1999). The effect of bee honey on the proliferative activity of human B- and T lymphocytes and the activity of phagocytes. Food Agric. Immunol..

[11] Khan R.U., Naz S., Abudabos A.M. (2017). Towards a better understanding of the therapeutic applications and corresponding mechanisms of action of honey. Environ. Sci. Pollut. Res. Int..

[12] Llor C., Moragas A., Ouchi D., Monfà R., Garcia-Sangenís A., Gómez-Lumbreras A., Pera H., Pujol J., Morros R. (2023). Effectiveness of antitussives, anticholinergics, and honey versus usual care in adults with uncomplicated acute bronchitis: A multiarm randomized clinical trial. Fam. Pr..

[13] Nazari-Bonab H., Jamilian P., Radkhah N., Zarezadeh M., Ebrahimi-Mameghani M. (2023). The effect of propolis supplementation in improving antioxidant status: A systematic review and meta-analysis of controlled clinical trials. Phytother. Res..

[14] Esposito C., Garzarella E.U., Bocchino B., D’Avino M., Caruso G., Buonomo A.R., Sacchi R., Galeotti F., Tenore G.C., Zaccaria V. (2021). A standardized polyphenol mixture extracted from poplar-type propolis for remission of symptoms of uncomplicated upper respiratory tract infection (URTI): A monocentric, randomized, double-blind, placebo-controlled clinical trial. Phytomedicine.

[15] Crişan I., Zaharia C.N., Popovici F., Jucu V., Belu O., Dascălu C., Mutiu A., Petrescu A. (1995). Natural propolis extract NIVCRISOL in the treatment of acute and chronic rhinopharyngitis in children. Rom. J. Virol..

[16] Khayyal M.T., El-Ghazaly M.A., El-Khatib A.S., Hatem A.M., De Vries P.J.F., El-Shafei S., Khattab M.M. (2003). A clinical pharmacological study of the potential beneficial effects of a propolis food product as an adjuvant in asthmatic patients. Fundam. Clin. Pharmacol..

[17] Buha I., Mirić M., Agić A., Simic M., Stjepanovic M., Milenkovic B., Nagorni-Obradovic L., Skodric-Trifunovic V., Ilic B., Popevic S. (2022). A randomized, double-blind, placebo-controlled study evaluating the efficacy of propolis and N-acetylcysteine in exacerbations of chronic obstructive pulmonary disease. Eur. Rev. Med. Pharmacol. Sci..

[18] Kolarov V., Kotur Stevuljević J., Ilić M., Bogdan M., Tusek B., Agic A., Dugajlic M., Veres K.T., Stevic S.K., Zvezdin B. (2022). Factorial analysis of N-acetylcysteine and propolis treatment effects on symptoms, life quality and exacerbations in patients with Chronic Obstructive Pulmonary Disease (COPD): A randomized, double-blind, placebo-controlled trial. Eur. Rev. Med. Pharmacol. Sci..

[19] Nault D., Machingo T.A., Shipper A.G., A Antiporta D., Hamel C., Nourouzpour S., Konstantinidis M., Phillips E., A Lipski E., Wieland L.S. (2024). Zinc for prevention and treatment of the common cold. Cochrane Database Syst. Rev..

[20] Careddu D., Pettenazzo A. (2018). *Pelargonium sidoides* extract EPs 7630: A review of its clinical efficacy and safety for treating acute respiratory tract infections in children. Int. J. Gen. Med..

[21] Bereznoy V.V., Riley D.S., Wassmer G., Heger M. (2003). Efficacy of extract of *Pelargonium sidoides* in children with acute non-group A beta-hemolytic *Streptococcus* tonsillopharyngitis: A randomized, double-blind, placebo-controlled trial. Altern. Ther. Health Med.

[22] Nöldner M., Schötz K. (2007). Inhibition of lipopolysaccharid-induced sickness behavior by a dry extract from the roots of *Pelargonium sidoides* (EPs 7630) in mice. Phytomedicine.

[23] Kolodziej H. (2011). Antimicrobial, Antiviral and Immunomodulatory Activity Studies of *Pelargonium sidoides* (EPs® 7630) in the Context of Health Promotion. Pharmaceuticals.

[24] Shulman S.T., Bisno A.L., Clegg H.W., Gerber M.A., Kaplan E.L., Lee G., Martin J.M., Van Beneden C. (2012). Infectious Diseases Society of America. Clinical practice guideline for the diagnosis and management of group A streptococcal pharyngitis: 2012 update by the Infectious Diseases Society of America. Clin. Infect. Dis..

[25] Sauve L., Forrester A.M., Top K.A. (2021). Group A streptococcal pharyngitis: A practical guide to diagnosis and treatment. Paediatr. Child Health.

[26] Baillie E.J., Merlo G., Magin P., Tapley A., Mulquiney K.J., Davis J.S., Fielding A., Davey A., Holliday E., Ball J. (2022). Antibiotic prescribing for upper respiratory tract infections and acute bronchitis: A longitudinal analysis of general practitioner trainees. Fam. Pract..

[27] Butler C.C., Simpson S.A., Dunstan F., Rollnick S., Cohen D., Gillespie D., Evans M.R., Alam M.F., Bekkers M.J., Evans J. (2012). Effectiveness of multifaceted educational programme to reduce antibiotic dispensing in primary care: Practice based randomised controlled trial. BMJ.

[28] Little P., Stuart B., Francis N., Douglas E., Tonkin-Crine S., Anthierens S., Cals J.W., Melbye H., Santer M., Moore M. (2013). Effects of internet-based training on antibiotic prescribing rates for acute respiratory-tract infections: A multinational, cluster, randomised, factorial, controlled trial. Lancet.

[29] Blair P.S., Young G.J., Clement C., Dixon P., Seume P., Ingram J., Taylor J., Horwood J., Lucas P.J., Cabral C. (2023). A multifaceted intervention to reduce antibiotic prescribing among CHIldren with acute COugh and respiratory tract infection: The CHICO cluster RCT. Health Technol. Assess..

[30] Kronman M.P., Gerber J.S., Grundmeier R.W., Zhou C., Robinson J.D., Heritage J., Stout J., Burges D., Hedrick B., Warren L. (2020). Reducing Antibiotic Prescribing in Primary Care for Respiratory Illness. Pediatrics.

[31] Cardinale F., Barattini D.F., Morariu Bordea M., Herteg D., Matei C.R. (2023). A Randomized, Open, Controlled Study to Evaluate the Efficacy and Safety of Pediaflù® (Dietary Supplement) Along with Standard of Care in Children with Acute Tonsillopharyngitis/Rhinopharyngitis versus Standard of Care Only V.1. https://www.protocols.io/view/a-randomized-open-controlled-study-to-evaluate-the-kqdg398oqg25/v1.

[32] Saint-Raymond A., Hill S., Martines J., Bahl R., Fontaine O., Bero L. (2010). CONSORT 2010. Lancet.

[33] Cardinale F., Barattini D.F., Sbrocca F., Centi A., Giuntini G., Morariu Bordea M., Herteg D., Rosu S., Matei C.R. (2024). The Effects of a Dietary Supplement (PediaFlu) Plus Standard of Care in Children with Acute Tonsillopharyngitis/Rhinopharyngitis: Protocol for a Randomized Controlled Trial. JMIR Res. Protoc..

